# Human lifestyle-associated factors modulate the gut resistome

**DOI:** 10.1128/msystems.01458-25

**Published:** 2026-04-07

**Authors:** Rahgavi Poopalarajah, Aashish R. Jha

**Affiliations:** 1 Genetic Heritage Group, Program in Biology, New York University Abu Dhabi 167632 , Abu Dhabi, United Arab Emirates; 2 Public Health Research Center, New York University Abu Dhabi 167632 , Abu Dhabi, United Arab Emirates; 3 Center for Genomics and Systems Biology, New York University Abu Dhabi 167632 , Abu Dhabi, United Arab Emirates; Monash University, Melbourne, Victoria, Australia

**Keywords:** antibiotic resistance, microbiomes, gut health, resistome

## Abstract

Antimicrobial resistance poses a serious threat to global public health in the 21st century. The human gut is a major reservoir of antimicrobial resistance genes and is strongly shaped by lifestyle factors linked to urbanization. Antibiotic use is widely known as a main driver of gut antimicrobial resistance; however, lifestyle encompasses other host and environmental determinants that also profoundly impact the gut resistome. These factors restructure gut microbiome composition and diversity, which, in turn, shape the abundance, persistence, and mobility of ARGs within the gut ecosystem. Lifestyle transitions along the urbanization gradient illustrate how antibiotic use, subsistence strategies, diet, agriculturally linked environmental exposures, host health, and global patterns of ARG distribution influence gut microbial diversity and ARG prevalence. Frequent antibiotic use in urban settings disrupts gut homeostasis and promotes resistome expansion. Transitions from traditional subsistence strategies to industrialized food systems are associated with dietary changes, such as reduced fiber intake, contributing to lower gut microbial diversity and increased ARG burden. Agrarian practices involving close human and livestock contact and antimicrobial use in animal husbandry facilitate ARG dissemination through the food chain. Host physiological factors and environmental exposures across diverse geographic regions additionally influence gut microbiome resilience and resistome composition. Integrating microbial community structure with ARG profiles provides insight into how lifestyle factors shape the gut resistome and influence ARG emergence and spread.

## INTRODUCTION

At the global scale, antimicrobial resistance (AMR) contributes to ~4.95 million deaths annually (1), and disproportionately burdens rapidly urbanizing regions such as the South-East Asia Region (WHO-SEAR), where population density, hygiene and sanitation conditions, and improper waste disposal exacerbate the problem (2–4). The gut microbiome harbors a collection of antimicrobial resistance genes (ARGs), which form the gut resistome (5). ARGs can be found in both pathogenic and non-pathogenic microorganisms in the gut, which confer resistance to antimicrobial molecules through intrinsic mechanisms, enzymatic inactivation or modification of antibiotics, mutations in chromosomally encoded antimicrobial target genes, or by acquisition and dissemination of ARGs (6–13). The ARGs in the gut can have serious implications for host health. Intestinal colonization by antibiotic-resistant pathogens, such as vancomycin-resistant *Enterococcus faecium* (VRE), *Klebsiella pneumoniae*, and *Escherichia coli,* can increase the risk of bloodstream infections and sepsis (14). ARGs can establish in the gut microbiome through ingestion of contaminated food and water, or via selective pressures such as antibiotic use, which drive their acquisition and persistence (15). Horizontal gene transfer (HGT) is facilitated by mobile genetic elements (MGEs) and promotes the spread of ARGs within gut microbial communities (11–13). At the same time, the gut microbiota can mitigate AMR through colonization resistance and competitive exclusion (16–18). The resident microflora occupies microbial colonization sites along the intestinal epithelium, limiting exogenous microbes, including antibiotic-resistant bacteria, from colonizing and infiltrating the gut barrier. Additionally, commensal microbes may inhibit the growth of antibiotic-resistant pathogens through immune modulation, nutrient depletion, and production of short-chain fatty acids (SCFAs) or bacteriocins (19). Together, ARG acquisition, dissemination, and microbial competition drive gut resistome composition.

**Fig 1:**
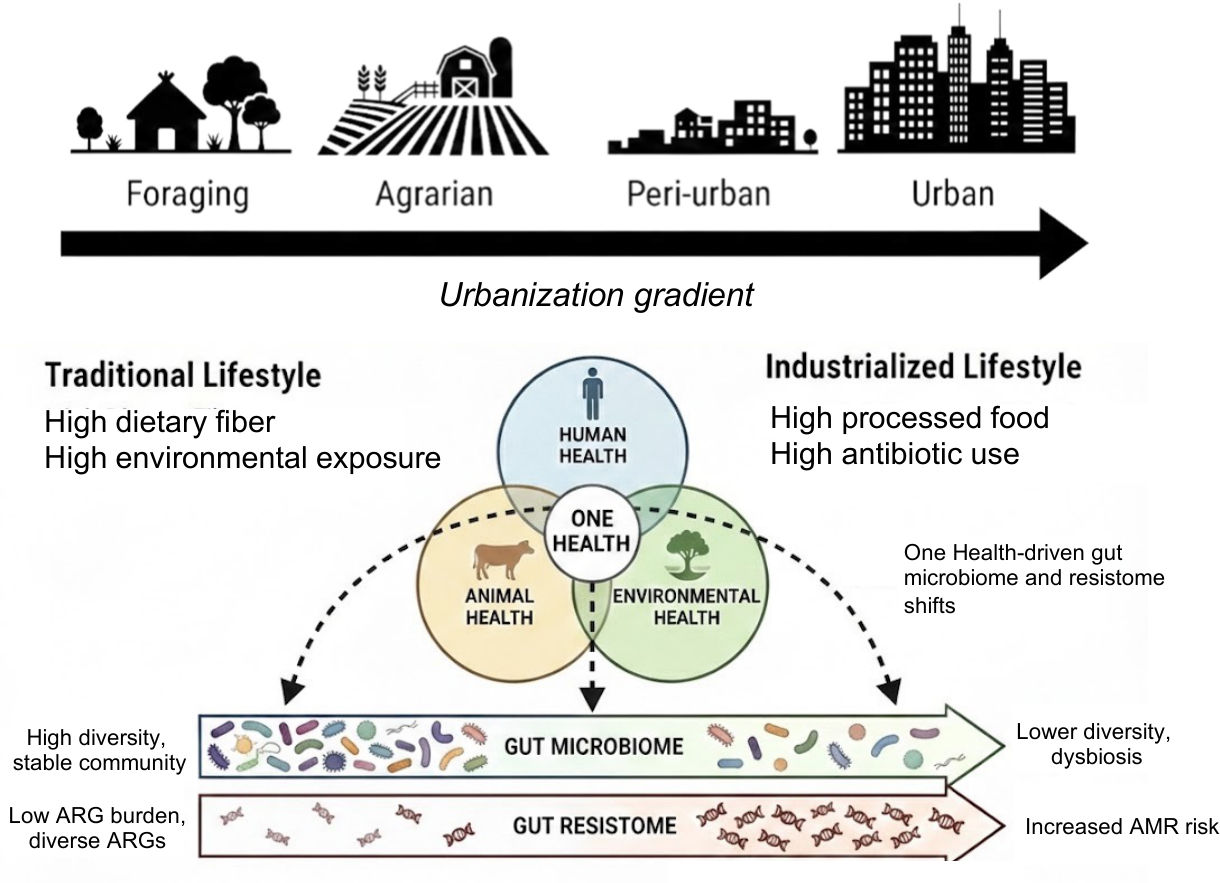
The gut microbiome and resistome across the urbanization gradient. Human populations span a continuum from foraging and agrarian to per-urban and urban lifestyles, which are shaped by factors such as diet, environmental exposure, sanitation, and antibiotic use. Traditional lifestyles are associated with high dietary fiber intake and frequent environmental and animal contact, while urban/industrialized lifestyles are characterized by increased consumption of processed foods, higher sanitation, and greater antibiotic exposure. These transitions are shaped by interconnected One Health influences across human, animal, and environmental health. Along this gradient, the gut microbiome shifts from a diverse and stable community toward reduced diversity and dysbiosis, while the gut resistome shows increasing abundance and altered composition of ARGs, reflecting increased AMR risk in more urbanized settings.

The gut microbiome, and consequently the gut resistome, are significantly impacted by urbanization (20–27) (Fig. 1). Several factors associated with urban lifestyles, such as diets rich in processed foods, highly sanitized environments that reduce pathogen burden, higher rates of birth by cesarean sections which limit vertical microbial transfer, and increased antibiotic use, contribute to gut microbiome perturbations and shape the gut resistome (28–30). While using antibiotics has long been recognized as the principal driver of AMR, growing evidence shows that additional host and environmental factors also contribute to AMR in the gut (31). These factors reflect subsistence strategies, referring to the dominant ways human populations obtain food and resources, such as foraging, small-scale farming, pastoralism, and industrial food systems, which shape human lifestyles and exposures (32). Lifestyle transitions from foraging or farming to increasingly industrialized and urbanized modes of living define the urbanization gradient that underpins this review.

**Fig 2:**
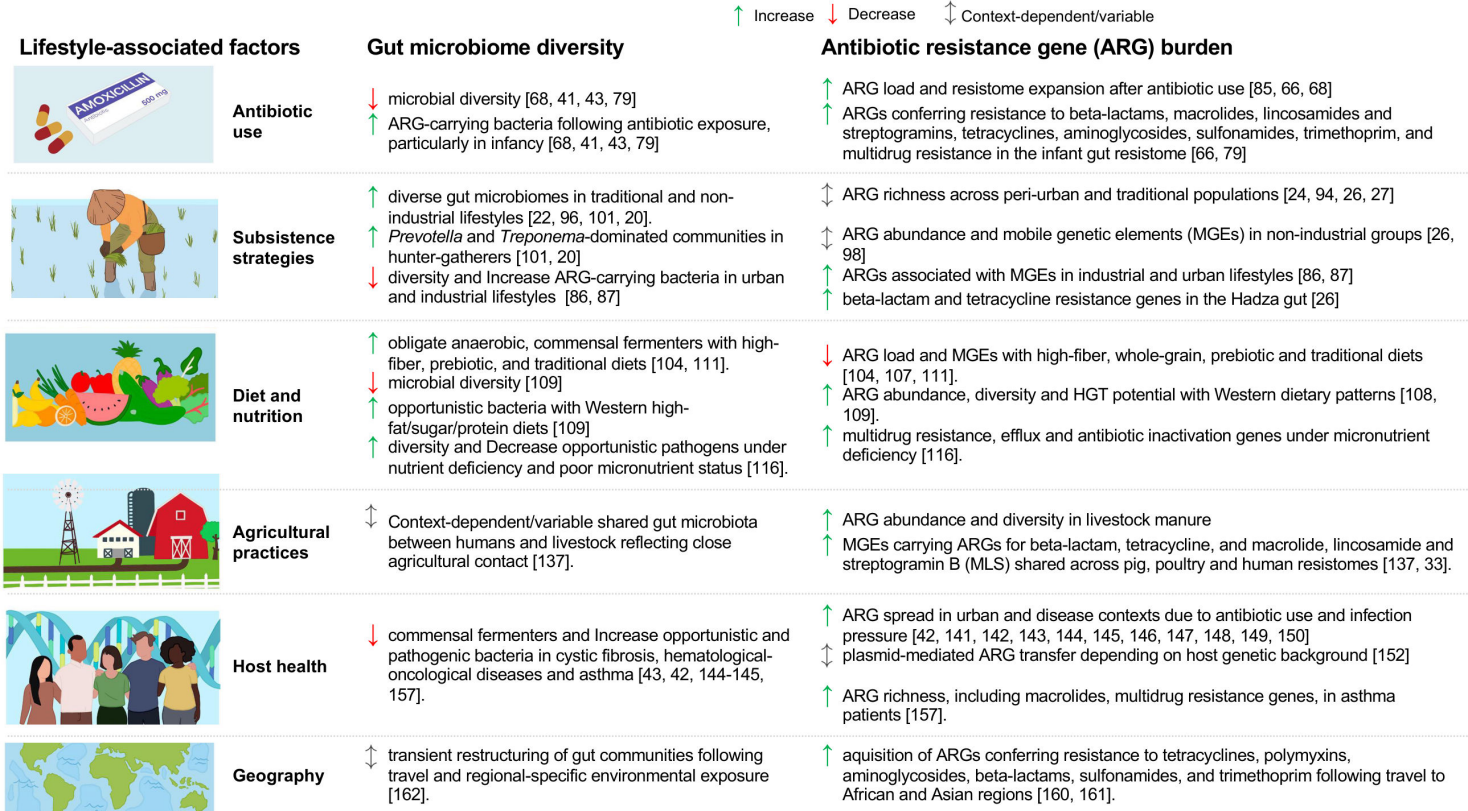
Lifestyle-associated factors, including antibiotic use, subsistence strategies, diet and nutrition, agricultural practices, host health, and geography, contribute to shifts in the gut microbiome and resistome.

From a One Health perspective, lifestyle-associated factors influence the gut resistome through interconnected human, animal, and environmental influences (33–36). Along the urbanization gradient, livestock practices, wildlife exposures, available water sources, and access to sanitation and household infrastructure differ markedly, shaping the reservoir of microbial taxa that can acquire, retain, and transmit ARGs (20–23, 25–27) (Fig. 2). For each lifestyle factor, we evaluate gut microbial composition, ARG diversity, and ARG abundance in the gut, while recognizing that relative abundance-based analyses cannot reliably distinguish shifts in microbial community structure from changes in total ARG burden, as demonstrated in low-biomass microbiome systems (37). Unless otherwise stated, ARG abundances are reported using relative abundance metrics derived from metagenomic sequencing and should be interpreted accordingly.

## GUT MICROBIOME COMPOSITION AND HGT SHAPE THE GUT RESISTOME

The gut resistome composition reflects underlying microbial community structure (38, 39). Low gut taxonomic diversity is broadly associated with higher resistome burden, and ARG distribution is closely linked to microbial phylogeny (7, 40, 41). Many clinically relevant ARGs are restricted to specific taxa. For example, beta-lactam resistance genes, CTX-M, KPC, IMP, NDM, and VIM, are enriched in *Pseudomonadota*, while *cepA*, *cblA*, *cfxA,* and *cfiA* beta-lactamase genes are linked to commensal *Bacteroides* (7). Similarly, glycopeptide resistance genes are predominantly detected in *Bacillota*, and many of the sulfonamide, trimethoprim, and fluoroquinolone ARGs are found in *Pseudomonadota* (42). Network analyses further indicate that a limited number of taxa, such as *Escherichia coli*, harbor the majority of ARG subtypes, which represent distinct gene variants within the same resistance classes, in the adult gut (40). Similar taxon-specific structuring is evident in early life, where increased *Pseudomonadota* abundance is associated with higher ARG burden and enrichment of *Enterobacteriaceae* including *E. coli* and *Klebsiella pneumoniae*, which are taxa known for plasmid-mediated HGT (41, 43, 44). Together, these observations reinforce the close relationship between microbial taxonomy and ARG carriage across life stages. Fungi, primarily yeasts such as *Candida albicans*, are normal inhabitants of the gut microbiome (45). Notably, yeast colonization has been negatively associated with ARG abundance, with yeast-positive samples exhibiting reduced copy numbers of 15 ARGs (45). A healthy gut microbiota is highly diverse and dominated by a small number of core microbial phyla (46). Disruptions to this balanced ecosystem can result in the expansion of opportunistic, ARG-carrying bacteria.

In the gut microbiome, HGT is mediated by MGEs, including plasmids and integrative and conjugative elements (ICEs) that enable conjugation, transposons that mobilize ARGs within and between genomes, bacteriophage-mediated transduction, and transformation via uptake of extracellular DNA (11, 12, 47, 48). Large-scale longitudinal analyses show that HGT is pervasive in the human gut microbiome (49). Specifically, 61.2% of metagenome-assembled genomes, representing 116 species, participate in at least one HGT event, yielding 7,581 transferred segments (49). Enrichment of adaptive functions, such as AMR, indicates strong positive selection in the gut environment. More than 150 HGT events involve tetracycline resistance gene transfer across multiple gut genera (49). ARG mobility is largely constrained by phylogeny and occurs predominantly within phyla (13, 50, 51). Although cross-phyla transfer is comparatively rare, it can occur within the gut microbiome, particularly between commensals and pathogens that inhabit the same gut niches, where close physical proximity, shared resources, and common selection pressures promote gene exchange. High-throughput chromatin conformation capture (Hi-C) and network-based approaches show that ARG exchange is concentrated among co-occurring taxa within individual gut communities, spanning both commensal and opportunistic bacteria (13). Commensal taxa within these communities harbor ARGs that are shared with opportunistic pathogens. For example, the *vanB* transposon originating from anaerobic bacteria in the gut, including *Eggerthella lenta* and *Clostridium innocuum,* has been identified in *Enterococcus faecium* isolates, demonstrating cross-phylum gene exchange in the gut (52). In addition, *Bacteroidota*, which dominate the gut microbiome, serve as major carriers of *tetQ* (tetracycline resistance) and *ermF*/*ermB* (macrolide-lincosamide-streptogramin B resistance) ARGs, including in individuals without recent antibiotic exposure (53). The high sequence identity (>99%) of *ermB* between gut *Bacteroidota* and pathogens such as *Clostridium perfringens*, *Streptococcus pneumoniae,* and *Enterococcus faecalis* supports ongoing commensal-pathogen HGT in the gut (53).

HGT among commensal taxa also contributes to ARG dissemination within the gut ecosystem. *Akkermansia muciniphila* has acquired *aph(6)-Id* (aminoglycoside resistance) and *sul2* (sulfonamide resistance) through HGT from many gut bacteria, including *Bacteroides* spp., *Bifidobacterium longum*, and several *Bacillota*, representing an evolutionary adaptation to the antibiotic-rich environment of the modern human gut (54). These ARGs are frequently associated with broad host-range plasmids and ICEs and are distributed across diverse gram-positive and gram-negative bacteria (55). Similarly, the tetracycline resistance gene *tet(W)*, initially identified adjacent to MGEs in a single *Bifidobacterium* lineage, is now widespread across the *Bifidobacterium* pan-genome, exemplifying how early MGE-mediated acquisition can lead to long-term persistence of ARGs in the gut (56). Collectively, these observations suggest the gut flora plays a central role in shaping resistome dynamics and ARG evolutionary trajectories (57).

## ANTIBIOTIC USE IN MODERN MEDICINE SELECTS FOR AMR BACTERIA IN THE GUT

Antibiotics are the main selective pressure driving the emergence and persistence of ARGs (58). Antibiotic exposure depletes beneficial gut bacteria, such as *Bifidobacterium* and *Faecalibacterium*, driving dysbiosis that is characterized by reduced microbial diversity and impaired colonization resistance, which, in turn, facilitates opportunistic pathogen expansion in the gut (59). Moreover, antibiotics can accelerate the spread of AMR via HGT, particularly at subinhibitory and environmentally relevant concentrations that stimulate conjugation and transformation through stress-induced physiological responses in bacteria (60, 61). Increased economic growth and urbanization have increased antibiotic consumption in both health and agriculture (62). The rates of antibiotic consumption are substantially higher in upper-middle-income countries, although misuse is more widespread in lower-middle-income countries (LMICs) due to limited healthcare access, poor sanitation, and unregulated medicine sales (63–65). In contrast, remote communities generally experience lower antibiotic exposure, particularly hunter-gatherer populations who rarely use therapeutic antimicrobials and experience minimal exposure through food sources (26).

In the context of rapid urbanization and rising antibiotic use, early-life antibiotic exposures have become increasingly common and can have profound consequences on the developing gut microbiome and contribute to the expansion of the gut resistome (66). In industrial settings, at least 40% of infants receive antibiotics before the age of 1 (67). The research shows that resistome burden is highest in the earliest weeks after birth and declines slowly after the first year of life (68). Preterm babies are particularly affected, as prophylactic antibiotic administration is routinely used to prevent neonatal complications (69, 70). Antibiotic treatment can perturb the infant gut resistome, especially in preterm infants. Reported outcomes range from long-lasting resistome alterations to transient changes that diminish with gut microbiome maturation (71–73). Modern obstetric practices further shape early resistome trajectories. Cesarean delivery typically involves intrapartum antibiotic prophylaxis, which disrupts early gut colonization, leading to reduced abundance of key anaerobic taxa such as *Bacteroidota* in the infants (74). In the context of antibiotic-driven dysbiosis and low microbial diversity, neonates have been reported to carry higher relative abundances of ARGs, although these differences may partly reflect compositional effects in low-diversity microbiomes (75, 76). The neonatal intestine also exhibits lower colonization resistance from the lack of established gut flora, which would allow for exogenous bacteria, including antibiotic-resistant pathogens, to readily colonize the gut (77). Together, the high frequency of antibiotic exposure during critical windows of microbiome assembly, and limited colonization resistance in early life, renders the infant gut particularly susceptible to resistome expansion in modern medical contexts.

Antibiotic impacts on the gut resistome vary widely across populations because they occur within broader ecological, microbial, and lifestyle contexts. Urbanization and westernization drive shifts in healthcare access, prescribing practices, and use of different antibiotic classes, introducing selective pressures that shape both ARG composition and persistence (78). Broad-spectrum antibiotics, for example, can exert long-lasting impacts on the gut resistomes of neonates with early-onset neonatal sepsis (66). In particular, amoxicillin and cefotaxime had the most profound effect on gut ARG composition and ARG profile in antibiotic-treated infants (66). Compared to untreated controls, ARGs *aac(6*'*)-aph(2), blaCMY-2, ermB, ermC,* and *mecA* were enriched in the infant gut following this antibiotic regimen. In other reports, use of penicillins increased beta-lactamase resistance genes and the abundance of *K. pneumoniae* (79). Similarly, sulfonamides, cephalosporins, and penicillins were linked to a higher prevalence of *tolC* (MDR efflux pump gene), correlating with increased relative abundance of *E. coli* (79). Using macrolides, cephalosporins, and penicillins was linked to higher levels of *tet32* (ribosomal protection protein) and *Ruminococcus gnavus* (79). These results suggest antibiotic use may selectively promote growth of ARG-harboring microbes or optimize ARG transfers between gut microbes via MGEs (60, 61). Broad-spectrum antibiotics are more commonly used in high-income countries, whereas access and regulation are more limited in lower-income regions (62). Nevertheless, studies from LMICs demonstrate antibiotic class-specific effects on infant gut microbiota diversity and ARG prevalence and richness (80). Exposure to broader-spectrum agents, such as azithromycin (a macrolide antibiotic) and amoxicillin (beta-lactam), has been associated with reduced gut microbial diversity and enrichment of macrolide and beta-lactam ARGs (81–83). In contrast, more targeted antibiotics such as cotrimoxazole selectively enriched trimethoprim and sulfonamide resistance genes, independent of treatment duration (84). Despite these observations, data from LMICs remain limited on long-term health consequences of antibiotic-driven gut microbiome perturbation and ARG burden. To summarize, antibiotics can substantially alter gut resistome profiles, but these effects are further shaped by multiple factors such as antibiotic class and treatment duration. These effects are additionally influenced by underlying microbiome composition, host physiology, co-exposures, and environmental conditions that collectively influence how the gut resistome changes over time (68, 85).

## GUT ARG PROFILES ARE STRUCTURED BY LIFESTYLE PRACTICES AND SUBSISTENCE STRATEGIES

Gut ARG profiles are strongly shaped by lifestyle practices and subsistence strategies along the urbanization gradient, although comparisons between industrial and non-industrial populations reveal nuanced and sometimes contrasting patterns. Urban and industrial populations have been shown to harbor increased relative abundances of antibiotic-resistant *Escherichia* and *Shigella*, reflecting a constriction of gut microbial diversity that favors ARG-carrying taxa enriched in MGEs under strong antibiotic selection in clinical and food-production settings (25–27, 86, 87). In contrast, *Prevotella* illustrates lifestyle-linked differences within a single lineage, including variation in abundance, strain composition, and ARG content across westernized and non-westernized groups (88). *Prevotella* isolates from Western cohorts from the United States and Europe carry more tetracycline resistance genes and exhibit higher prevalence of antibiotic inactivation mechanisms than isolates from non-Western populations, including cohorts from Madagascar, Tanzania, Peru, and India (89). However, regional studies further demonstrate that ecological context can modulate lifestyle-driven resistome patterns (24). In China, less industrialized western regions showed higher overall gut ARG richness and abundance than industrialized eastern regions despite lower antibiotic pressure (24). Both regions harbored high levels of tetracycline and macrolide-lincosamide-streptogramin resistance genes (24). While beta-lactam resistance was enriched in eastern gut microbiomes, gut-associated multidrug resistance genes and pathogenic bacteria were more prevalent in the western regions (24). These results indicate that pathogen burden can reshape lifestyle-driven resistome gradients. Moreover, an urban cohort from Kazakhstan, recruited from a major medical center within a mixed urban-peri-urban-rural landscape, showed gut microbiome compositions distinct from highly industrialized populations in the United States, China, Denmark, Sweden, and Spain (90). Interestingly, despite the high antibiotic consumption and elevated abundances of typical ARG-carrying bacteria, the Kazakh cohort exhibited a lower overall gut ARG burden than industrialized Western groups (90). This suggests that lifestyle-associated environmental exposures, microbial transmission dynamics, or pathogen load may shape gut ARG composition even under strong antibiotic selection.

Peri-urban areas that are formed through rural-urban transitions occupy an intermediate position between traditional rural and fully urbanized environments, where intensifying human activity increasingly creates hotspots for ARG dissemination (91). These settings are closely tied to subsistence agriculture, involving frequent contact with livestock, soils, and environmental discharge (92, 93). In Lima, Peru, individuals from a peri-urban, industrializing community had a higher gut resistome burden than Peruvian hunter-gatherers, rural agriculturalists (Peru, El Salvador), and US-based individuals (94). This elevated ARG burden in the gut likely reflects unregulated antibiotic use combined with repeated exposure to ARG-carrying pathogens from both agricultural runoff and nearby urban environments. Multi-habitat analyses further revealed human feces-derived ARGs in street sewage, influent, and treated effluent that can be reintroduced into surrounding environments (94). In contrast, rural agriculturalist communities showed extensive ARG sharing among humans, livestock, latrines, and agricultural soils, with poultry-associated soils acting as key reservoirs. Together, these results demonstrate that industrializing peri-urban areas can harbor disproportionately high ARG loads, driven by consistent ARG flow between human, animal, and environmental niches along the urbanization gradient.

Before agriculture, Paleolithic hunter-gatherers interacted closely with natural ecosystems, wildlife, and soils that served as reservoirs of ancient ARGs (95). Studies of the Hadza show that their gut resistomes include soil-associated ARGs such as tetracycline and beta-lactam resistance genes, also found in medieval dental calculus (26). This highlights the potential for ARGs to be transmitted from the environment to humans through foraging. Hadza resistomes have been reported to contain greater ARG diversity and fewer MGEs than Western resistomes, which is consistent with lower antibiotic exposure and extensive contact with environmental microbes (26). Yet, findings across Hadza studies vary due to methodological and ecological differences. One study reported that the Hadza gut contained ARGs that were distinct from but less diverse than ARGs in industrial gut resistomes, whereas other metagenomic analyses observed high total ARG abundance and greater overall ARG diversity in the gut microbiomes of Hadza and other non-industrial groups compared with US populations (27, 96). Similarly, the Yanomami gut and oral microbiomes share high sequence similarity with ARGs found in industrialized populations despite minimal urban exposure (97). Many of these genes conferred resistance to later-generation antibiotics, reflecting the ancient and ubiquitous nature of ARGs. Comparisons of paleofaeces with pre-industrial and modern samples reveal enrichment of tetracycline resistance genes and MGEs compared to Paleolithic samples (98), likely reflecting the strong antibiotic-driven selection pressures that emerged in the post-antibiotic era. In remote Arctic populations, heavy metals transported through the marine food web exert environmental selection that maintains ARGs in the gut, in the absence of substantial antibiotic use (99, 100). These results illustrate that lifestyle-linked environmental exposures can shape the gut resistome of individuals in remote communities with minimal direct antibiotic pressure.

Comparisons across human and ape gut microbiomes demonstrate that lifestyle is a strong driver of resistome composition in both humans and non-human primates (27). Captive chimpanzees and gorillas cluster more closely with humans than with wild apes across taxonomy, gene families, and ARG profiles. Captive apes exhibit up to 34-fold higher ARG abundance and 5-fold higher ARG richness than wild apes, driven largely by tetracycline and beta-lactam resistance genes (27). In contrast, wild apes living in close proximity to non-Western human populations harbor markedly lower ARG abundance and richness, with resistomes dominated by aminoglycoside resistance genes likely linked to environmental exposures (27). Overall, these results indicate that lifestyle-mediated interactions with soil, water, wildlife, pathogens, and agricultural environments shape gut resistomes. Across the urbanization gradient, these dynamics reflect both ancient ARG reservoirs and modern antibiotic selection pressures.

## DIETARY AND NUTRITIONAL INFLUENCES ON GUT RESISTOME DYNAMICS ACROSS THE URBANIZATION GRADIENT

Dietary habits vary across traditional, transitioning, and urbanized lifestyles and strongly influence gut microbiomes and resistomes. Traditional subsistence diets, exemplified by the Hadza of Tanzania, include wild, foraged foods such as berries, fiber-rich tubers, baobab fruit, honey, and wild meat (101, 102). These diets are rich in complex plant carbohydrates and contrast with urban industrialized diets, which are characterized by simpler sugars and frequent exposure to synthetic compounds. Microbial metabolic activity varies with dietary composition and contributes to resistome structure (103). High soluble fiber intake (~8–10 grams/day) is associated with lower ARG loads and increased abundance of SCFA-producing obligate anaerobes, offering insights into how traditional diets rich in soluble fiber may help mitigate AMR (104, 105). SCFAs can restructure microbial communities and inhibit transfer of multi-drug resistant plasmids between pathogenic *E. coli* strains (106). Moreover, fiber supplementation reduces the abundance of ARGs across several antibiotic classes and MGEs in the pig colon (107). These effects are often attributed to enhanced SCFA production and reduced HGT, although many observations rely on relative abundance data and may reflect compositional shifts driven by fiber-fermenting taxa rather than absolute reductions in ARG load.

In contrast, western diets low in fiber and high in animal protein, sugar, starch, and fat are associated with greater gut ARG diversity and abundance. Diets rich in poultry and pork correlate with higher rates of antibiotic-resistant infections in older adults (108). In an American cohort, prevalence of the aminoglycoside resistance gene *aph(3′)* was inversely correlated with fiber intake and calorie consumption (104). High fiber intake was associated with less facultative anaerobes from *Enterobacteriaceae* and *Streptococcaceae* families, more obligate anaerobes from the family *Clostridiaceae*, and reduced gut resistome burden (104). Increased ARG abundance was also linked to lower ARG alpha-diversity and reduced gut microbial diversity. High-sugar, high-fat, and high-protein diets independently promote ARG prevalence in the gut over time and are linked to microbiome shifts, altered metabolites, and gut inflammation, which can enhance ARG transfer through increased bacterial cell membrane permeability and oxidative stress (109). However, diet-associated inflammation and microbiome restructuring do not contribute to significant, long-term differences in the gut resistome.

In a Dutch cohort, long-term dietary practices, including omnivorous, pescatarian, vegetarian, and vegan diets, had minimal impact on the gut microbiome and resistome (110). Total ARG abundance, dominant ARG classes (tetracyclines, macrolides, beta-lactams, aminoglycosides, and phenicols), and overall ARG diversity remained largely consistent across diets (110). The ARGs *Isa(C)* and *tet(L)* showed differential abundance between meat and seafood consumers but were detected at low frequencies in gut resistomes. In contrast, *tet(X*) was more prevalent in meat-based diets, while overall gut microbiome diversity was maintained across all dietary groups (110). By comparison, targeted dietary interventions such as whole grains, prebiotics, and traditional medicinal foods were associated with reductions in ARG types, defined as distinct resistance gene families, and total ARG counts in the gut (111). Decreases in gut ARGs coincided with decreased abundances of ARG-carrying bacteria, including *Klebsiella*, *Enterobacter,* and *Escherichia* species (111). These interventions enriched carbohydrate-fermenting taxa such as *Bifidobacteria* and *Lactobacilli*, highlighting dietary fiber as a key modulator of ARG propagation. In addition to dietary composition, nutritional status contributes to variation in gut microbiome development and resistome burden.

Undernutrition (which includes stunting, wasting, and underweight) is a leading cause of death among children under 5 years of age in LMICs (112). In 2022, undernutrition in SEAR remained high, with 30.5% of children stunted, 14.3% wasted, and 26% underweight, all exceeding global averages (113). Poor dietary intake disrupts gut microbiome development, promoting dysbiosis, inflammation, and expansion of the gut resistome (114–116). Micronutrient deficiencies in zinc, folate, iron, and vitamins A and B12 are associated with expansion of opportunistic bacteria, including *Klebsiella*, *Shigella, Escherichia*, and *Campylobacteria* (116). These taxa show positive correlations with antibiotic efflux and inactivation mechanisms. In micronutrient-deficient mice, ARGs encoding low membrane permeability and altered antibiotic targets were enriched, conferring resistance to multiple antibiotic classes including fluoroquinolones, macrolides, penams, fosfomycin, diaminopyrimidines, glycylcyclines, nitrofurans, and tetracyclines (116). Elevated oxidative stress in low micronutrient mice correlated positively with multidrug efflux pumps (*emeA* and *erfA*) and modified antibiotic targets (*isaA* and *dfrE*) and contributed to proliferation of opportunistic bacteria in the gut (116). These data highlight the importance of adequate micronutrient intake in maintaining gut microbiome stability and limiting AMR.

## AGRICULTURAL SYSTEMS FUNCTION AS KEY INTERFACES FOR ARG DISSEMINATION

Agrarian production systems and practices are central to a One Health framework because food links soil, plant, animals, and humans, serving as a major route of exposure to environmental microbes, pathogens, and ARGs (117). Agricultural soils act as major reservoirs of ARGs that can be transmitted through the soil-plant-food continuum. Global metagenomic analyses reveal that agricultural soils are enriched for ARGs that are commonly found in clinical pathogens and gut-associated microbes, underscoring soil as a critical interface for ARG exchange (118). These transmission routes intersect with widespread antibiotic use in agriculture, creating strong selective pressures that promote ARG persistence and spread.

In developing countries, agricultural antibiotic use is high, and industrialization has fueled misuse, leaving antimicrobial residues in food and the environment and accelerating the global spread of resistance (119–121). Livestock antibiotic consumption is projected to rise nearly 70% by 2030, and global use is concentrated in a small number of countries (122). In 2013, China was the world’s largest consumer of veterinary antibiotics, using over 162,000 tons, of which almost half was incorporated into livestock feed (123). By 2017, China accounted for 45% of total global antibiotic use in animals. Collectively, China, Brazil, the United States, Thailand, India, Iran, Spain, Russia, Mexico, and Argentina accounted for 75% of antibiotics used in animals worldwide, representing regions encompassing roughly half of the global population (124).

Within livestock and poultry food production systems, antibiotics are applied for disease treatment and prevention and growth promotion purposes (125). Other antimicrobial compounds are also used as feed additives, preservatives, or disinfectants to maintain hygiene along the food supply chain (126, 127). Together, these practices impose strong selective pressures that favor the persistence and spread of ARGs across agricultural systems. The misuse of critically important antibiotics like macrolides and polymyxins in farming contributes substantially to global AMR (128). A notable example is the plasmid-mediated colistin resistance gene *mcr-1*, which was first detected and selected for in food animal systems before its subsequent emergence in human clinical isolates (129), highlighting cross-sector emergence of resistance to a last resort antibiotic. As a consequence of this extensive and often indiscriminate antibiotic use in livestock systems, pig slaughterhouses have emerged as key hotspots of ARGs (130–132). Metagenomic studies of pig processing facilities show that these environments accumulate clinically relevant ARGs over time, with increased ARG diversity, co-occurrence of MGEs, and evidence of HGT on food-contact surfaces and drains following the onset of processing activities (132). Similarly, high levels of clinically relevant ARGs have been detected in pig manure from commercial pig farms across multiple Chinese provinces (Beijing, Zhejiang, and Fujian), particularly genes conferring resistance to macrolides, cephalosporins, aminoglycosides, and tetracyclines (131). Notably, despite the limited use of aminoglycosides in these farms, aminoglycoside resistance genes increased by up to 10,000-fold in manure, suggesting strong co-selection of ARGs on MGEs driven by exposure to other antibiotics and metals (131, 133). Together, these findings highlight slaughterhouse and livestock-processing environments as important non-clinical settings for ARGs and MGE accumulation across animals and the environment.

These agricultural reservoirs are further connected to humans through contact with farmed animals, facilitating microbial exchange involving zoonotic taxa and reinforcing agriculture as a key interface for AMR emergence across interconnected systems (134–136). ARGs are frequently shared between food animals and humans, reflecting common environmental reservoirs and interconnected microbial gene pools. In a comparative analysis of 145 human and 87 pig fecal metagenomes, 27 ARGs were detected across all gut resistomes (137). The most abundant ARGs conferred resistance to tetracycline and macrolide-lincosamide-streptogramin B antibiotics. Several of these ARGs were linked to well-characterized MGEs carried by high-abundance gut commensals, especially *Bacteroidetes* and *Firmicutes*, which dominate the intestinal microbiota of both humans and pigs (137). For example, the ICE ICExyXB1A, carrying the tetracycline resistance gene *tetQ* and macrolide resistance gene *ermF*, in addition to the conjugative transposon CTnBst harboring *bla*OXA-347, was predominantly detected in human gut microbiota. In contrast, pig gut microbiota were enriched in CTnGERM1, which carries the macrolide efflux genes *mefA* and *melA*, and the transposon Tn4555 and conjugative transposon CTnHyb, both encoding *cfxA5*, a beta-lactamase conferring resistance to cephamycins. Moreover, the mobilizable transposon MTnSag1 and the putative composite MGE CMGEYY060816, which were associated with the lincosamide resistance gene *InuC* and tetracycline resistance gene *tet40*, respectively, were present in both human and pig microbiota, indicating a widespread distribution across host species (137). Although these MGEs are largely carried by commensal gut bacteria, many, including members of the Tn916/Tn1545 family of conjugative transposons, are widely documented in clinically relevant pathogens (138).

Similarly, a study of poultry production systems identified 73 clinically relevant ARGs shared between humans and chickens (33). Closely related *E. coli* strains were detected in more than 75% of both human and animal gut samples (33). Although strain-level ARG associations were not directly assessed, near-identical MGEs carrying ARGs in human and chicken gut resistomes implicate shared environments as hotspots for MGE-mediated ARG exchange. Additionally, proximity to farming activities has been shown to impact the human gut resistome (139). A 30.8% overlap was observed between human and swine resistomes, and ARGs originating from air, soil, and groundwater were detected in human gut microbiota (139). Also, veterinary students residing on a swine farm collectively acquired 270 environmental ARGs within 3 months (136). Approximately 25% of gut-associated ARGs co-localized with putative MGEs, supporting a role for MGEs in environmental to human transfer (136). In these works, high-abundance MGEs in the gut microbiota contribute to ARG dissemination across interconnected human, animal, and environmental resistomes within a One Health context. On this basis, AMR surveillance strategies should extend beyond clinical isolates to encompass environmental and agricultural reservoirs.

## URBANIZATION-DRIVEN HOST HEALTH AND DISEASE-ASSOCIATED SHIFTS IN THE GUT RESISTOME

The WHO describes urban health as the impact of city environments on human health (140). Poor housing, sanitation, and waste management increase disease burden, driving antibiotic use that perturbs the gut microbiome and accelerates gut resistome expansion (42, 140). Disease-associated resistomes are therefore important for antibiotic treatment decisions because ARGs provide selective advantages under antibiotic pressure and commonly co-occur with virulence genes that facilitate microbial survival and persistence in the host (141–143). In cystic fibrosis, chronic antibiotic exposure leads to gut microbiota dysbiosis, characterized by depletion of fermentative commensals (i.e., *Faecalibacterium prausnitzii* and *Ruminococcus bromii*) and enrichment of ARG-harboring pathobionts such as *E. coli* and *Enterococcus spp*. (144, 145). Similarly, diarrhea-associated microbiomes show increased abundance of ARGs conferring resistance to cephalothin, piperacillin, aztreonam, spiramycin, ceftriaxone, and gentamicin antibiotic classes following repeated antibiotic treatment (146, 147). These changes occurred alongside shifts in gut microbial community structure, including the loss of obligate anaerobes and the expansion of facultative ARG-rich *Enterobacteriaceae* adapted to inflamed gut environments. Together, these disease contexts link recurrent antibiotic exposure to gut microbiome restructuring and enrichment of ARGs in the gut. During the COVID-19 pandemic, antibiotics were prescribed to 75% of patients in North America and Europe and 80% of patients in LMICs, while bacterial co-infections were identified in fewer than 10% of cases (148, 149). Antibiotic-treated COVID-19 patients showed pronounced gut resistome expansion compared to untreated individuals (150). A threefold increase in ARG abundance was observed, and elevated ARG levels persisted for up to 6 months, following infection (151). Evidence from hematological-oncological cohorts demonstrates that broad-spectrum antibiotics such as cotrimoxazole selected for plasmid-born ARGs in *Pseudomonadota*, while ciprofloxacin treatment was associated with reduced MGEs across multiple taxa, highlighting opportunities to optimize treatment regimens to limit selection for mobile resistance determinants (42).

Host health reflects the combined influence of genetics and environmental factors, yet their effects on the gut resistome have not been fully elucidated. Emerging evidence indicates that host genetic factors can mediate ARGs transfer via MGEs (152). For example, the inbred C3H/HeN mouse strain, widely used in studies of cancer, inflammation, and cardiovascular disease (153), exhibited distinct gut resistome dynamics compared to the wild-type 129S6/SvEv strain (152). Both strains were colonized with the altered Schaedler flora (ASF). However, 129S6/SvEv ASF mice showed higher levels of transconjugants carrying plasmid pCVM29188_146, encoding streptomycin and tetracycline resistance genes in feces than C3H/HeN ASF mice (152). These results illustrate that host genetic background can influence plasmid transfer efficiency in the gut under defined microbiota conditions. However, gene transfer depends on recipient availability and plasmid host range. More research is needed to understand host factors regulating conjugation in the gut. In addition to genetic influences, disease states associated with urbanized environments provide important contexts in which gut resistome restructuring becomes clinically relevant.

Asthma prevalence is higher in industrialized societies and has been linked to urban environmental factors such as air pollution and limited green space (154). Interestingly, traditional farming practices are associated with reduced asthma and allergy risk, likely through greater exposure to endotoxin (lipopolysaccharide from gram-negative bacteria) in organic dust from crop and livestock farming environments that modulates immune responses (155). The hygiene hypothesis links reduced microbial exposure and antibiotic use in Western lifestyles to gut microbiome perturbations that increase asthma risk (156). Early-life gut ARG abundance is associated with asthma-linked microbiome profiles. Both infants and adults with asthma exhibit higher gut ARG richness than healthy individuals, despite no significant differences in total ARG load (43, 157). This increase in richness is linked to shifts in the relative abundance of ARG-carrying taxa and enrichment of macrolide resistance (*ermF*, *ermB*, and *ermA*) and multidrug efflux pump genes (*smeB*, *mdtO*, and oqxA) (157). In addition, co-occurrence analyses reveal distinct ARG-virulence gene combinations associated with *K. pneumoniae* and *E. coli* in asthma (157). These data demonstrate that disease and antibiotic exposure drive changes in gut microbiome composition, ARG abundance, and virulence profiles. Human mobility then facilitates the dissemination of these resistomes across populations.

## THE GLOBAL DISTRIBUTION OF ARGs REFLECTS HUMAN LIFESTYLES

Industrialization and urban growth have increased antibiotic use and, together with human migration and globalization, accelerated the global dissemination of ARGs across diverse environments and populations (158). Country-specific variation in gut resistome composition has been linked to differences in antibiotic consumption across populations (159). Spanish gut microbiomes contained the highest number of ARGs compared with Danish and U.S. samples, and US gut microbiomes were enriched for macrolide, lincosamide, and streptogramin resistance, which are linked to animal agriculture (159). Overall, gut ARG prevalence strongly correlates with region-specific patterns of human antibiotic use.

International travel is a well-established risk factor for ARG dissemination (160–162). Short-term travel to Africa and Asia contributed to ARG enrichment in the gut resistomes of Dutch travelers (160). Analysis of 190 fecal samples identified 56 distinct ARGs spanning 11 of 20 AMR classes after travel, including genes mediating drug efflux and altered antibiotic targets (160). Travel to Southeast Asia was linked to increased detection of region-specific ARGs, notably *tetX* (tetracycline inactivation) and *mcr-1*, the latter detected in 18 of 52 travelers (160). Exchange students traveling to the Indian peninsula or Central Africa carried bacteria with ARGs encoding resistance to tetracyclines (*tetB*), aminoglycosides [*aph(3″)-Ib* and *aph(6)-Id*], beta-lactams (DHA and TEM beta-lactamases), sulfonamides (*sul2*), and trimethoprim (*dfrA*) (161). Moreover, extended-spectrum beta-lactamase (ESBL)-producing *E. coli* was identified in 12 of 18 travelers following visits to the Indian peninsula (161). Additional longitudinal studies documented high prevalence of multidrug-resistant and ESBL-producing *Enterobacterales* following travel to high-risk regions, including acquisition of *CTX-M* beta-lactamases after travel to Sub-Saharan Africa, *mcr-1* after travel to Peru, and plasmid-mediated quinolone resistance (PMQR) genes upon return from sub-Saharan Africa (162). To conclude, international travel facilitates the redistribution of ARGs, including resistance determinants shaped by local antibiotic use, highlighting the roles of global mobility and regional lifestyle practices in AMR spread.

## THE GUT MICROBIOME IS A KEY TARGET IN CIRCUMVENTING ARG TRANSMISSION

Lifestyle modulates the gut microbiome, which, in turn, influences the repertoire of ARGs within populations. For this reason, microbiome-directed interventions may alter gut AMR profiles (163, 164). Probiotics and fecal microbiota transplantation (FMT) can restore microbial composition and function by enhancing colonization resistance and suppressing opportunistic, ARG-harboring taxa, leading to reduced ARG abundance (165, 166). Probiotics are live microorganisms that promote gut homeostasis through improved gut barrier integrity and immune function (167). In small cohorts of adults receiving probiotics following colonoscopy, probiotic administration was associated with decreased ARG diversity and abundance, particularly in luminal samples from individuals permissive to gut colonization by probiotic strains (168). Similarly, preterm infants receiving the probiotic formulation FloraBABY, containing *Bifidobacterium breve*, *Lactobacillus rhamnosus*, *B. bifidum*, *B. longum* subsp*. infantis*, and *B. longum* subsp*. longum*, exhibited limited ARG diversity over a 5-month period (70). FMT in patients with recurrent *Clostridium difficile* infection has likewise been linked to shifts toward *Bacteroidota* and *Bacillota* and concomitant reductions in ARG abundance and diversity (169). In renal transplant recipients, FMT replaced multidrug-resistant organisms with antibiotic-susceptible strains of the same species (170). In particular, ESBL-producing strains were replaced by non-ESBL counterparts susceptible to trimethoprim-sulfamethoxazole and nitrofurantoin antibiotic classes, likely driven by strain-level competition (170). However, evidence supporting probiotics and FMT to mitigate AMR is mixed. Probiotic strains carrying ARGs may increase gut resistome burden (168, 171). Probiotic use following antibiotic treatment has been associated with elevated ARG abundance due to delayed microbiome recovery and expansion of endogenous ARG-harboring taxa, such as *Blautia* spp., carrying multidrug efflux and vancomycin resistance genes (*vanSD*) with co-occurring MGEs, suggesting potential HGT (168). More research is needed to define the impact of probiotics on ARG dissemination and to evaluate the risks associated with live-strain therapeutics.

The gut microbiome is a complex microbial ecosystem with interaction networks that may be harnessed to counter AMR in pathogenic gut microbes (172, 173). A recent study systematically assessed community-level responses to antimicrobials using a synthetic gut community of 32 healthy species exposed to 30 drugs (174). Across 1,823 drug-species combinations, community-mediated protection was observed in 68%, 47% and 23% of cases at low, medium, and high drug concentrations, respectively, and cross-sensitivity occurred in 4%, 8%, and 11% of combinations, respectively (174). Community-mediated protection likely arises from microbial biotransformation or sequestering of antibiotics intracellularly, thereby lowering drug exposure for neighboring taxa (175). These polymicrobial interactions are less studied in the gut but may help explain discrepancies between *in vitro* and *in vivo* antimicrobial responses and inform strategies to improve AMR treatment outcomes (176).

Microbiome-directed modulation of the gut resistome has potential implications for One Health efforts aimed at limiting the emergence and dissemination of AMR. Alterations in microbial community structure can influence ARG abundance, mobility, and persistence, thereby affecting transmission across human, animal, and environmental reservoirs. In this context, strategies that account for gut microbial ecology may complement antibiotic stewardship and environmental control approaches for AMR mitigation.

## FINAL PERSPECTIVES

Antibiotic resistance is shaped by lifestyle changes associated with urbanization, including dietary shifts, widespread antibiotic use, intensive livestock production, host health status, and environmental exposures. These factors influence the abundance, composition, and mobility of ARGs, although many of their effects remain incompletely understood. Growing evidence indicates that preserving or restoring gut microbial diversity can modulate resistome structure, highlighting the gut microbiome as a potential target for strategies aimed at limiting ARG emergence and spread, including dietary interventions and microbiome-directed approaches (163).

Addressing AMR requires a One Health perspective that recognizes the interconnected roles of human practices, environmental exposures, and microbial ecology in shaping resistance dynamics (177, 178). Future AMR research and surveillance should more fully integrate lifestyle, environmental, and ecological drivers that are underrepresented relative to clinical and antibiotic-focused studies. Integrating these dimensions will be critical for developing effective, preventative strategies to reduce the AMR burden across populations and ecosystems.
